# Development and Application of a Duplex Droplet Digital Polymerase Chain Reaction Assay for Detection and Differentiation of EP402R-Deleted and Wild-Type African Swine Fever Virus

**DOI:** 10.3389/fvets.2022.905706

**Published:** 2022-06-06

**Authors:** Junhai Zhu, Weijun Jian, Yifan Huang, Qi Gao, Fei Gao, Huahan Chen, Guihong Zhang, Ming Liao, Wenbao Qi

**Affiliations:** ^1^College of Veterinary Medicine, South China Agricultural University, Guangzhou, China; ^2^African Swine Fever Regional Laboratory of China (Guangzhou), Guangzhou, China; ^3^Key Laboratory of Zoonoses, Ministry of Agriculture and Rural Affairs of the People's Republic of China, Guangzhou, China; ^4^National and Regional Joint Engineering Laboratory for Medicament of Zoonoses Prevention and Control, National Development and Reform Commission of the People's Republic of China, Guangzhou, China; ^5^Key Laboratory of Zoonoses Prevention and Control of Guangdong, Guangzhou, China

**Keywords:** African swine fever virus, droplet digital polymerase chain reaction, laboratory diagnosis, sensitive detection, gene-deleted strain differentiation

## Abstract

African swine fever (ASF) is a highly fatal porcine disease caused by the African swine fever virus (ASFV), and resulting in huge economic losses across the globe. ASF has been raging in China for 3 years, and recently EP402R-deleted ASFV strains emerged, showing sub-acute or chronic symptoms in pigs and providing novel difficulties to monitor and control the disease as EP402R-deleted strains possess no hemadsorption (HAD) ability. In addition, the gene deletion virus with low viral load is prone to results retest or false negative due to the high cycle threshold (Ct) value under the current real-time polymerase chain reaction (PCR) detection method. Thus, a new method is needed to detect and distinguish wild strains and gene-deleted viruses. In this study, a duplex droplet digital polymerase chain reaction (ddPCR) assay based on the ASFV B646L and EP402R genes was established and showed good linearity (R^2^ > 0.99). The limit of detection for duplex ddPCR was 52 copies per reaction and 8.6 copies per reaction for B646L and EP402R, respectively. No cross-reaction with other porcine viruses [classical swine fever virus (CSFV), porcine reproductive and respiratory syndrome virus (PRRSV), porcine epidemic diarrhea virus (PEDV), porcine parvovirus (PPV), Japanese encephalitis virus (JEV), and porcine circovirus type 2 (PCV2)] was identified by this assay. In addition, 44 ASFV-suspicious clinical samples as well as EP402R-deleted ASFV were tested in parallel by duplex real-time PCR and ddPCR, indicative of a higher sensitivity which belonged to the duplex ddPCR assay. In summary, this is the first time that duplex ddPCR assay has been successfully developed to provide an efficient method to detect and differentiate ASFV wild-type and gene-deleted strains.

## Introduction

African swine fever (ASF) is a highly fatal porcine disease caused by the African swine fever virus (ASFV), leading to huge economic losses and food security threats worldwide. The ASFV genome is between 171 and 193 kbp, encoding more than 150 proteins and forming a mature ASFV virion with a multilayered structure and overall icosahedral morphology (1). The first ASF outbreak in China was reported in August 2018, and sequencing result revealed a genotype II ASFV (2, 3). Currently, ASF has raged in Asia and spread over 16 countries including Korea (4), Indonesia (5), and Vietnam (6). Pigs intramuscularly inoculated with epidemic strain Pig/HLJ/18 demonstrated acute clinical symptoms including fever, loss of appetite, depression, lethargy, respiratory distress, vomiting, and death in all of the pigs (3). However, EP402R gene (encoding CD2v protein)-deletion strains were isolated from China in 2020, which caused sub-acute or chronic disease, and persistent infection in pigs (7). It is worth mentioning that EP402R gene-deletion strains lost hemadsorption (HAD) ability, and hence conventional HAD assay could not detect EP402R gene-deletion virus infection. EP402R-deleted vaccine candidates, on the other side, also caused sub-acute or chronic symptoms in pigs (8, 9). No ASFV vaccine candidate has been licensed, but once the gene-deleted vaccine is successfully developed and commercialized in the future, it would be widely used for the control of ASF worldwide. Therefore, a novel method is needed to distinguish EP402R-deleted ASFV strains or vaccines developed in the future.

On account of the complex structure of the ASFV virion and the limited knowledge on ASFV infection mechanisms and immunity, no commercial vaccines and therapeutic drugs have been developed yet. Therefore, early phase monitoring of diseases, culling of infected animals, and strict biosecurity measures are dominating methods of disease prevention and control at this stage. A mass of detection methods for ASFV, such as virus isolation, HAD assay, fluorescent antibody detection, polymerase chain reaction (PCR), and enzyme-linked immunosorbent assay (ELISA) has been established and applied (10–14). Among them, real-time polymerase chain reaction (real-time PCR) is widely applied for early detection of pathogens in clinical samples due to rapid, highly sensitive, convenient and specific features. However, real-time PCR methods rely on external standards, standard curves, and the cycle within the amplification process that the reporter dye signal exceeds a threshold [cycle threshold (Ct) value].

Droplet digital polymerase chain reaction (ddPCR) is a novel pathogen detection technology that enables absolute quantification of nucleic acids without using the standard samples. In a ddPCR assay, the amplification reaction is divided into thousands of independent partitions containing either a single target molecule or none at all in each individual reaction mixture. After amplification in partitioned reactions to the endpoint with positive and negative partitions is counted, the number of DNA targets per partition and the copy number in the original sample are calculated by Poisson's Law. On comparing with real-time PCR, one of the golden standard methods for ASFV detection, ddPCR has advantages of absolute quantification, less affected by sample inhibitors, and better detection of low-copy-number samples (15). Methods of ASFV detection by ddPCR with a high degree of linearity and specificity have been established (16, 17). However, current ASFV ddPCR strategies only detect single viral gene and possess no ability to distinguish gene-deleted virus from wild strains.

In this study, based on the B646L gene (conserved and essential gene encoding the major capsid protein p72) and the EP402R gene (encoding the viral hemagglutinin protein CD2v), a probe-based duplex ddPCR was developed to distinguish wild-type or gene-deleted strains ASFV infection. The duplex ddPCR assay in this study showed superior characteristics, such as highly sensitivity, low detection limit, and gene-deleted ASFV strains differentiation capability, and thus possessed the potential to monitor both wild-type ASFV and EP402R-deleted strains infection.

## Materials and Methods

### Recombinant Standard Plasmid and Virus Genome Preparation

This sentence has been rewritten as “The full length of B646L gene (1,941 base pairs) and EP402R gene (1,083 base pairs) were obtained from ASFV strain GZ201801 (GenBank accession number MT496893.1) by PCR and cloned into pMD18-T vector, which named pB646L and pEP402R. Primers were showed in Table 1. GZ201801-ASFV genome was extracted from inactivated cell-cultured ASFV supernatant by the AxyPrep Body Fluid Viral DNA/RNA Miniprep Kit (Axygen; Corning Inc., Corning, NY, United States). Genomes of classical swine fever virus (CSFV), porcine reproductive and respiratory syndrome virus (PRRSV), porcine epidemic diarrhea virus (PEDV), porcine parvovirus (PPV), Japanese encephalitis virus (JEV), porcine circovirus type 2 (PCV2), ΔEP402R/ΔMGF360-505-ASFV, ΔEP402R-ASFV, and ΔMGF360-505-ASFV are preserved in the South China Agricultural University (18).

**Table 1:** Primers used for the recombinant standard plasmids construction.

**PrimerQ11**	**Sequence**(5^**′**^-3^**′**^**)**	**Position**
pB646L-F	TTAGGTACTGTAACGCA	103607–103623
pB646L-R	ATGGCATCAGGAGGA	105547–105533
pEP402R-F	ATGATAATACTTATTTTTTTAATAT	73383–73407
pEP402R-R	TTAAATAATTCTATCTACGT	74446–74465

### Primer and Probe Design

To design the generalized primers and probe of EP402R genes, 37 ASFV genomes were obtained from GenBank and used for multiple sequence alignments by DNAMAN. The upstream primer sequences (5′-ATGTTGAAGAAATAGAAAGTC-3′) were fully matched to the target gene of all viral genomes, except for individual gene mutations in the downstream primer (5′-GACTGTAAGGCTTAGGAA-3′) and probe [5′-(HEX)-TGACACCACTTCCATACATGAACCA-(BHQ1)-3′] (Table 2). The TaqMan real-time PCR detection of the ASFV B646L gene was carried out according to the Office International des Epizooties (OIE)-recommended procedure described previously (19). Moreover, the designed primers and probes were fully matched to genotype II (dominating genotype in China) and genotype I (emerging genotype in China) ASFV. In duplex detection, the probe for the B646L gene was labeled with the 5′-reported dye FAM and the 3′-quencher MGB, while the probe for the EP402R gene was labeled with the 5′-reported dye HEX and the 3′-quencher BHQ1. All primers and probes were purchased from Sangon Biotech (Shanghai, China).

**Table 2:** Primer and probe of EP402R were aligned with 37 African swine fever virus (ASFV) strains.

**ASFV isolate**	**Probe**	**Anti-sense primer**
M78 (genotype V)	TGACACCACTTC**T**ATACATGAACCA	GACT**A**TA**T**GGCTTAGGAA
MK-200 (genotype V)	TGACACCACTTC**T**ATACATGAACCA	GACT**A**TA**T**GGCTTAGGAA
Ndjassi-77 (genotype I)	TGACACCACTTCCATACATGAACCA	GACT**A**TAAGGCTTAGGAA
Spencer (genotype I)	TGACACCACTTCCATACATGAACCA	GACT**A**TAAGGCTTAGGAA
L-50 (serotype I)	TGACACCACTTCCATACATGAACCA	GACT**A**TAAGGCTTAGGAA
TS7 (genotype X)	TGACACCACTTC**T**ATACATGAACC**G**	GACTGTAAGGCTTAGGAA
691/88 (serotype IV)	TGACACCACTTC**T**ATACATGAACC**G**	GACTGTAAGGCTTAGGAA
Katanga (genotype I)	TGACACCACTTCCATACATGAACCA	GACT**A**TAAGGCTTAGGAA
Krasnodar_2012/dom (serotype VIII)	TGACACCACTTCCATACATGAACCA	GACTGTAAGGCTTAGGAA
Tver_2012/wb (serotype VIII)	TGACACCACTTCCATACATGAACCA	GACTGTAAGGCTTAGGAA
Uganda (genotype X)	TGACACCACTTC**T**ATACATGAACC**G**	GACTGTAAGGCTTAGGAA
Volgograd_2012/dom (serotype VIII)	TGACACCACTTCCATACATGAACCA	GACTGTAAGGCTTAGGAA
TSP80 (genotype X)	TGACAC**T**ACTTC**T**ATACATGA**G**CC**G**	GACTGTAAGGCTTAGGAA
Estonia 2014 (genotype II)	TGACACCACTTCCATACATGAACCA	GACTGTAAGGCTTAGGAA
CN201801 (genotype II)	TGACACCACTTCCATACATGAACCA	GACTGTAAGGCTTAGGAA
Wuhan 2019-1 (genotype II)	TGACACCACTTCCATACATGAACCA	GACTGTAAGGCTTAGGAA
Wuhan 2019-2 (genotype II)	TGACACCACTTCCATACATGAACCA	GACTGTAAGGCTTAGGAA
ASFV-wbBS01 (genotype II)	TGACACCACTTCCATACATGAACCA	GACTGTAAGGCTTAGGAA
CADC_HN09 (genotype II)	TGACACCACTTCCATACATGAACCA	GACTGTAAGGCTTAGGAA
CN/2019/InnerMongolia-AES01 (genotype II)	TGACACCACTTCCATACATGAACCA	GACTGTAAGGCTTAGGAA
DB/LN/2018 (genotype II)	TGACACCACTTCCATACATGAACCA	GACTGTAAGGCTTAGGAA
Pig/HLJ/2018 (genotype II)	TGACACCACTTCCATACATGAACCA	GACTGTAAGGCTTAGGAA
BA71V (genotype I)	TGACACCACTTCCATACATGAACCA	GACTGTAAGGCTTAGGAA
ASFV/Timor-Leste/2019/1 (genotype II)	TGACACCACTTCCATACATGAACCA	GACTGTAAGGCTTAGGAA
ASFV/pig/China/CAS19-01/2019 (genotype II)	TGACACCACTTCCATACATGAACCA	GACTGTAAGGCTTAGGAA
ASFV/Ulyanovsk 19/WB-5699 (genotype II)	TGACACCACTTCCATACATGAACCA	GACTGTAAGGCTTAGGAA
China/2018/AnhuiXCGQ (genotype II)	TGACACCACTTCCATACATGAACCA	GACTGTAAGGCTTAGGAA
CN/2019/InnerMongolia-AES01 (genotype II)	TGACACCACTTCCATACATGAACCA	GACTGTAAGGCTTAGGAA
Georgia_2007-1 (genotype II)	TGACACCACTTCCATACATGAACCA	GACTGTAAGGCTTAGGAA
Georgia_2008-1 (genotype II)	TGACACCACTTCCATACATGAACCA	GACTGTAAGGCTTAGGAA
GZ201801 (genotype II)	TGACACCACTTCCATACATGAACCA	GACTGTAAGGCTTAGGAA
HLJ-2018 (genotype II)	TGACACCACTTCCATACATGAACCA	GACTGTAAGGCTTAGGAA
Ken05-Tkl (genotype X)	TGACACCACTTC**T**ATACATGAACCA	GACTGTAAGGCTTAGGAA
Ken06-Bus (genotype X)	TGACAC**T**ACTTCCATACATGAACCA	GACTGTAAGGCTTAGGAA
L60 (genotype I)	TGACACCACTTCCATACATGAACCA	GACTGTAAGGCTTAGGAA
NHV (genotype I)	TGACACCACTTCCATACATGAACCA	GACTGTAAGGCTTAGGAA
OURT88/3 (genotype I)	TGACACCACTTCCATACATGAACCA	GACTGTAAGGCTTAGGAA

*The bold letters indicate the mutated bases*.

### Duplex ddPCR and Real-Time PCR Amplification Conditions

For duplex ddPCR assays, 20 μl of solution contained 10 μl of ddPCR supermix (Bio-Rad, Hercules, CA, USA), 2 μl of template, 900 nM primer and 500 nM probe at final concentration. The mixtures were then emulsified automatically with 70 μl droplet generator oil (QX-200 droplet generator; Bio-Rad), and 40 μl mixed droplets were transferred to a 96-well-reaction plate to heat seal at 180°C for 5s. The ddPCR cycling conditions were as follows: 95°C predenaturation for 10 min, followed by 40 cycles of denaturation at 94°C for 30s and annealing at 53.9°C for 1 min, and 1 cycle of enzyme inactivation at 98°C for 10 min. Finally, the plate was placed into a droplet reader (QX-200 droplet reader; Bio-Rad). For duplex real-time PCR assays, a 20 μl reaction mixture was made containing 10 μl of 2×AceQ real-time PCR Probe Master Mix (Vazyme Nanjing, Jiangsu, China), 0.4 μl of each primer (final concentration of 400 nM), 0.2 μl of each probe (final concentration of 200 nM), and 2μl of DNA template. The duplex real-time PCR was performed on the LightCycler96 Real-Time PCR System with cycling conditions as follows: 95°C for 5 min, followed by 40 cycles of 95°C for 10 sand 60°C for 30 s.

### Sensitivity and Specificity of ddPCR and Real-Time PCR

To evaluate the sensitivity of the primers and probes of the B646L gene and EP402R gene, gradient-diluted pB646L and pEP402R plasmid were used as templates and tested by ddPCR and real-time PCR. Briefly, copy numbers of the standard plasmids of pB646L and pEP402R were calculated based on the following formula:


$$\begin{array}{l} copy\;number\;\left(\text{copies}/\text{μL}\right)=\frac{\text{A}260\;\left(\text{ng}/\text{μL}\right)\times 10^{-9}\times 6.02\times 10^{23}}{\text{DNA length }\left(\text{bp}\right)\times 660} \end{array}$$

These two methods were then performed in parallel using the same standard plasmid as the template after 10-fold serial dilutions. To evaluate the specificity of the primers and probes of the B646L gene and EP402R gene, genomes of ASFV and other six common swine viruses (CSFV, PRRSV, PEDV, JEV, PPV, and PCV2) were used as templates and tested by duplex ddPCR with ASFV-specific primers and probes.

### Clinical Sample Detection

To compare the diagnostic specificity and identify the ability to distinguish between wild strains and gene-deletion viruses of duplex ddPCR and real-time PCR assay, a total of 44 pig blood samples collected from suspected ASFV-infected pigs were processed for DNA extraction and evaluated by duplex ddPCR and real-time PCR. EP402R-positive samples (inactivated GZ201801 and ΔMGF360-505-ASFV) and EP402R-negative samples (water, ΔEP402R-ASFV, and ΔEP402R/ΔMGF360-505-ASFV) were set at the same time.

## Results

### Optimization of the Duplex ddPCR Assay

**Figure 1:**
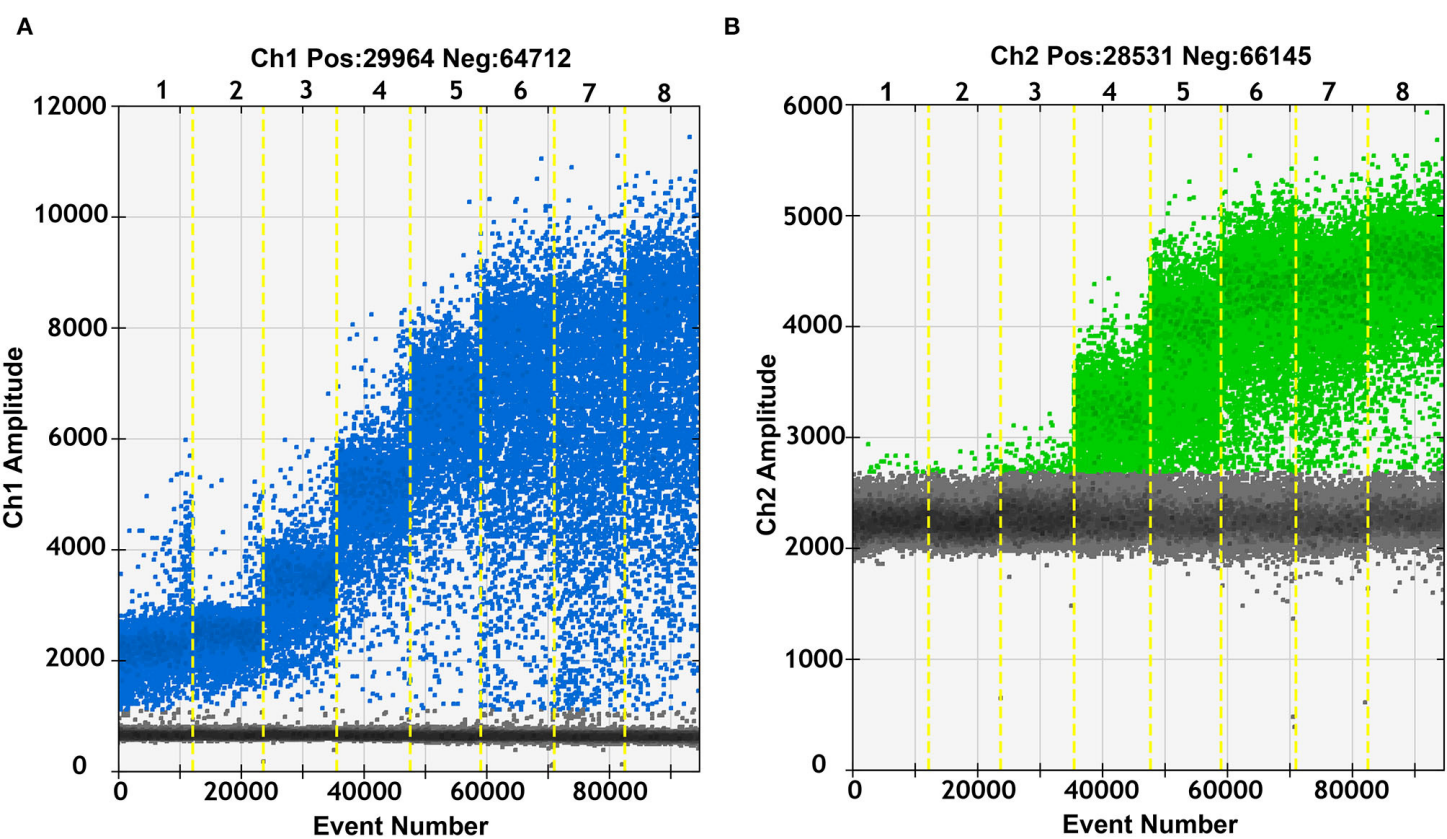
Annealing temperature optimization of duplex droplet digital polymerase chain reaction (ddPCR) assay. The duplex ddPCR assay for pB646L **(A)** and pEP402R **(B)** was optimized with different annealing temperatures and the fluorescence amplitude of amplification determined the optimal reaction condition. The annealing temperature gradient (lanes 1–8) was 60, 59.4, 58.3, 56.3, 53.9, 52, 50.7, and 50°C, respectively.

To validate the optimum annealing temperature of the duplex ddPCR assay, pB646L and pEP402R were used as templates and a gradient annealing temperature was set at 60, 59.4, 58.3, 56.3, 53.9, 52, and 50.7°C. Blue droplets and green droplets were shown as positive droplets for B646L and EP402R, respectively, while black droplets were negative droplets (Figure 1). For the primers and probe of the B646L gene, positive droplets were detected under all annealing temperature conditions but were differentiated well above 53.9°C. As for the primers and probe of the EP402R gene, positive droplets were differentiated well below 53.9°C. Therefore, an annealing temperature of 53.9°C was determined for the duplex ddPCR assay.

### Linearity and Limit Detection of Duplex ddPCR and Real-Time PCR Assay

**Figure 2:**
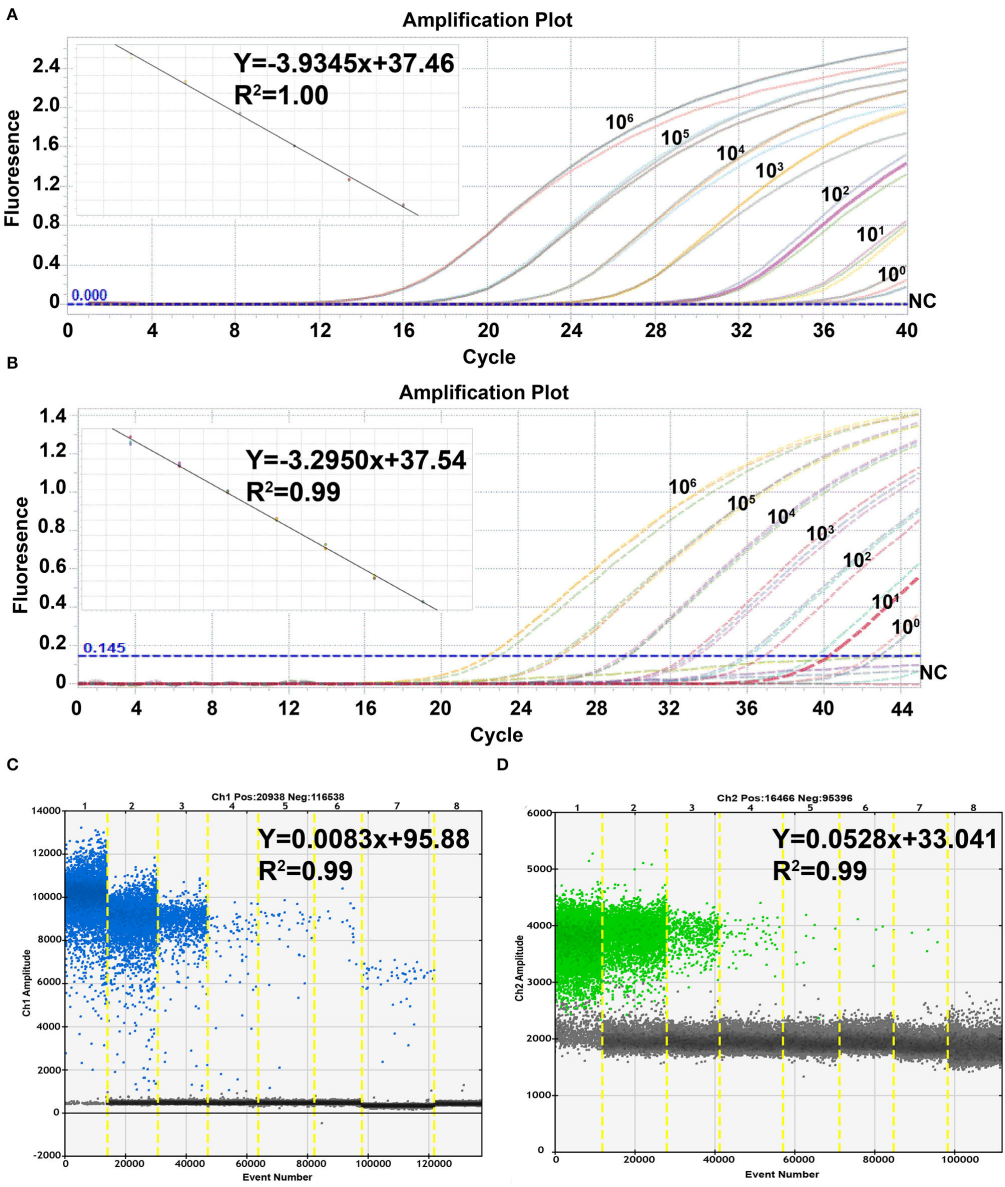
Sensitivity of the duplex real-time PCR and the ddPCR assay. The duplex real-time PCR assay for pB646L **(A)** and pEP402R **(B)** was evaluated at concentrations of 10_6_-10_0_ copies /μl template and nuclease-free water as negative control (NC). The duplex ddPCR assay for pB646L **(C)** and pEP402R **(D)** was evaluated at the same concentration condition and the template plasmid concentrations gradient (lanes 1–8) was 10^6^, 10^5^, 10^4^, 10^3^, 10^2^, 10^1^ and 10^0^ copies /μl, and NC.

To evaluate the linearity, sensitivity, and efficiency of the quantification of the ASFV duplex real-time PCR and ddPCR, 10-fold dilution standard plasmids of pB646L and pEP402R with concentrations ranging from 10^6^ to 10^0^ copies/μl were used as templates and tested by duplex real-time PCR and ddPCR. The standard curve of the duplex real-time PCR was plotted using the LightCycler96 Real-Time PCR System (ROCHE). As shown in Figures 2A,B, both duplex real-time PCR and ddPCR have a strong linear correlation (R^2^ > 0.99) between Ct values and the corresponding copy numbers of pB646L and pEP402R. The standard curves of pB646L and pEP402R were plotted with slopes of−3.9345 and−3.2950, respectively. The lowest detectable concentration of pB646L and pEP402R was 10 copies/μl in duplex real-time PCR assay but 1 copy/μl in duplex ddPCR assay (Figures 2C,D). In addition, the actual concentration value was revised as 52 copies/reaction and 8.6 copies/reaction by Poisson distribution for B646L and EP402R, respectively. These results indicated that the primers and probes used in the ddPCR assay possessed higher linearity, sensitivity, and efficiency, and lower limit detection compared to real-time PCR.

### Specificity of the Duplex ddPCR

**Figure 3:**
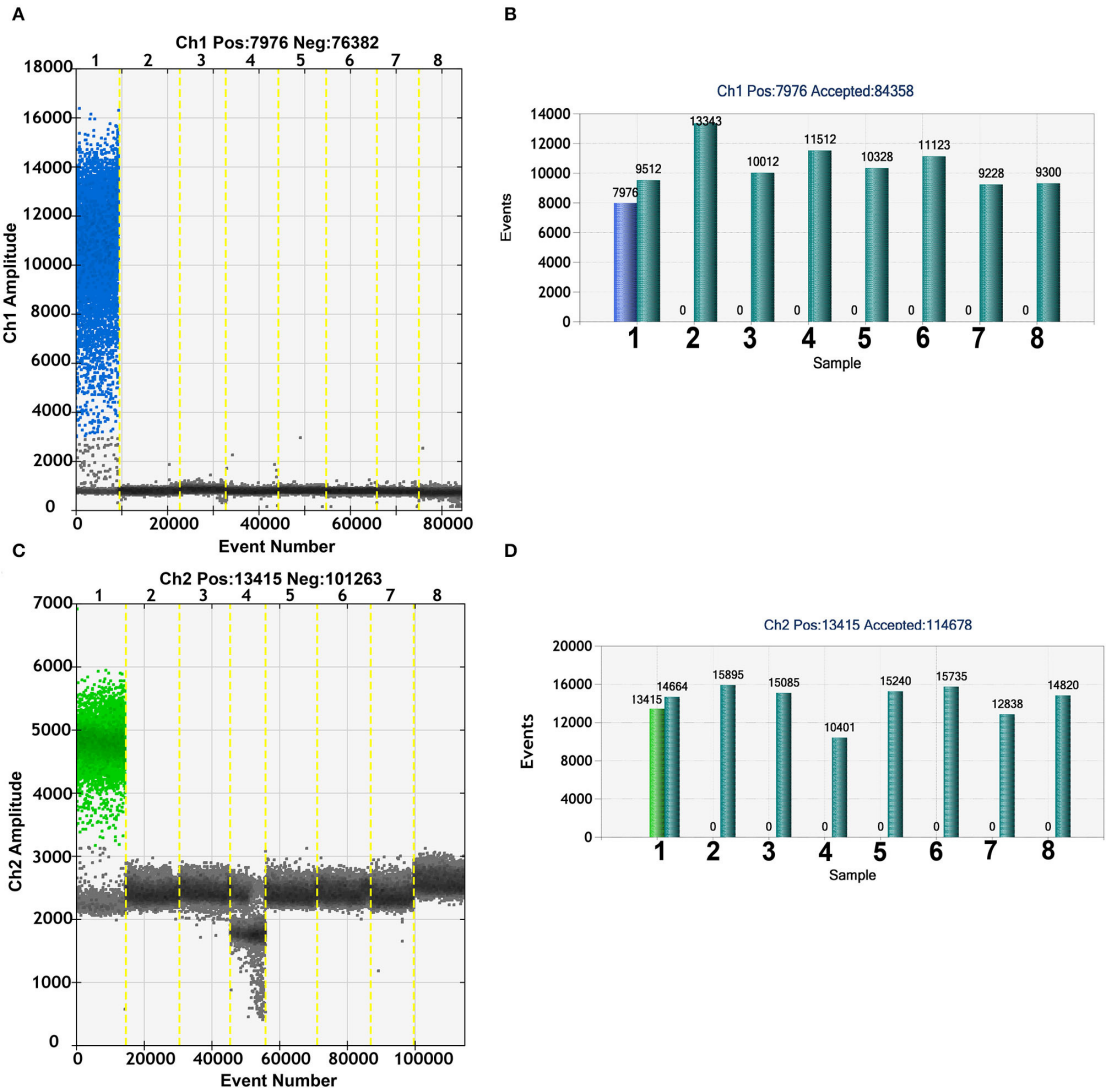
Specificity of the duplex ddPCR assay. The fluorescence amplitude of African swine fever virus (ASFV) (lane1) B646L **(A)** and EP402R **(C)** amplification was observed without cross-reactivity with CSFV, PRRSV, PEDV, JEV, PPV, PCV2 (lanes 2–7), and NC (lane 8). The ratio of B646L **(B)** and EP402R **(D)** for classical swine fever virus (CSFV), porcine reproductive and respiratory syndrome virus (PRRSV), porcine epidemic diarrhea virus (PEDV), porcine parvovirus (PPV), Japanese encephalitis virus (JEV), porcine circovirus type 2 (PCV2) (lanes 2–7), and NC (lane 8) positive events to total partitions was 0.

To evaluate the specificity of the duplex ddPCR assay, ASFV, CSFV, PRRSV, PEDV, JEV, PPV, and PCV2 were used as templates and tested by duplex ddPCR with primers and probes of B646L and EP402R. As shown in Figures 3A,C, no cross-reactivity was detected. Moreover, the positive events of ASFV B646L and EP402R molecules in other swine disease genome were zero (Figures 3B,D). These results demonstrated that both duplex ddPCR and real-time PCR assay possessed high specificity.

### Application of the Duplex ddPCR Assay in Clinical Samples

Forty-four swine blood samples as well as EP402R-positive samples (inactivated GZ201801 and Δ MGF360-505R-ASFV) and EP402R-negative samples (water, ΔEP402R-ASFV, and ΔEP402R/Δ MGF360-505R-ASFV) were tested by the established duplex ddPCR and real-time PCR. Duplex ddPCR detected 44 FAM and HEX signals in all ASFV-suspicious swine blood samples (44/44, 100%) (Supplementary Table S1). However, duplex real-time PCR demonstrated 42 samples showing FAM fluorescence curves (42/44, 95%) and 41 samples showing HEX fluorescence curves (41/44, 93%). Moreover, no HEX fluorescence signal was detected in either duplex ddPCR assay or duplex real-time PCR assay. Clinical samples of B646L or EP402R showed negative results by real-time PCR, which were also detected as very low nucleic acid concentration in ddPCR, indicating low viral loads in these samples.

## Discussion

African swine fever virus is a severe contagious swine disease. Strict biosecurity measures and slaughter are the main means of prevention and remediation before commercial vaccines and antiviral drugs were authorized. It caused huge economic losses, especially for a country like China that has a large-scale pig industry. CD2v, encoding by the EP402R gene, is required for protection against the challenge of the homologous ASFV strain. Several EP402R-deleted live attenuated vaccine candidates have demonstrated that CD2v is associated with virus replication and virulence *in vivo*, and EP402R-deleted strains can confer protection against homologous and heterologous ASFV strains (8, 9, 20, 21). In addition, ASFV strains, HLJ/HRB1/20 and HeB/Q3/20, demonstrated deletion in the EP402R gene, causing consequent loss of protein function (7). Thus, it is critical to develop an efficient detection method to differentiate not only EP402R-deleted but also mutated wild-type viruses from wild-type virus.

The PCR-based detection method is widely used in pathogen detection due to the swift, accurate, and efficient characteristics. Multifarious PCR-based detection methods have been established and applied to ASFV detection, such as real-time PCR, droplet digital PCR, loop-mediated isothermal amplification (LAMP) assay, recombinase polymerase amplification (RPA) assay, and CRISPR/Cas12a assay (16, 19, 22–25). Among them, ddPCR achieves theoretical single-molecule amplification through extreme dilution, and then uses PCR amplification and Poisson distribution to calculate the original concentration of the sample (26). Compared with real-time PCR, ddPCR possesses higher detection sensitivity without the requirement of standard products and standard curves. Meanwhile, end-point PCR signal count detection does not depend on the Ct value, and can effectively overcome the influence of PCR inhibitors. A ddPCR assay was developed based on the TaqMan probe targeting the ASFV K205R gene, showing a high degree of linearity and specificity and a 10-fold greater sensitivity compared with real-time PCR (16). Afterwards, a nanofluidic chip dPCR (cdPCR) was designed on the QuantStudio 3D digital PCR platform, which showed a 33-times higher limit detection compared with real-time PCR approved by OIE (17). Although several multiplex real-time PCR assays were developed to differentiate gene-deleted and wild-type viruses, existing ddPCR methods detected a single ASFV gene and possessed no ability to differentiate gene-deleted strains (27–29).

In this study, we developed and evaluated a duplex ddPCR assay targeting the B646L gene and EP402R gene. Two signals (FAM and HEX) were generated to represent the B646L and the EP402R gene, respectively. The B646L gene, which is a conserved region for wild-type and gene-deleted viruses, was selected as a reference gene to distinguish ASFV from other viruses. Detection of the FAM signal indicated the presence of ASFV nucleic acid, whether it was wild-type ASFV or EP402R-deleted ASFV. However, only FAM signal was detected implying that the EP402R was deleted, and further verification, sequencing for example, should be made to verify whether it is a vaccine candidate or mutant strain. The digital PCR result on 44 pig blood samples showed that no EP402R deletion virus was found, but samples with extreme low viral load demonstrated false negative results by real-time PCR assay. In addition, EP402R-deleted virus strains previously characterized (ΔEP402R/ΔMGF360-505-ASFV and ΔEP402R-ASFV) were detected as zero positive droplet by established duplex ddPCR assay, indicating a good application in EP402R-deleted and wild-type ASFV differentiation. A triplex real-time PCR assay targeting B646L gene, MGF_360-14L gene, and EP402R gene demonstrated a similar limit detection compared with the duplex ddPCR (27). Also, the limit detection of duplex ddPCR was similar with that of cdPCR assay established (30.1995 copies per reaction for B646L gene) (17). However, duplex ddPCR assay can only distinguish between wild virus strains and EP402R-deleted strains, which may lead to false test results when detecting other gene-deleted strains. Therefore, protocol-based multiple-cluster ddPCR assay is permissible to be developed in the future to differentiate other mutations associated with attenuated ASFV phenotypes from wild-type virus (30).

In summary, a specific, sensitive, and accurate duplex ddPCR assay to simultaneously detect ASFV B646L and EP402R was first established, which could distinguish EP402R-deleted viruses and wild-type viruses.

## Data Availability Statement

The raw data supporting the conclusions of this article will be made available by the authors, without undue reservation.

## Author Contributions

JZ and WQ: conceptualization, WJ: methodology, YH, QG, FG, and HC: data curation. JZ: writing—original draft preparation. WQ: writing—review and editing. GZ, ML, and WQ: funding acquisition. All authors contributed to the article and approved the submitted version.

## Funding

This work was funded by the Key-Area Research and Development Program of Guangdong Province (No. 2019B020211003), National Natural Science Foundation of China (No. 31941014), Project of Swine Innovation Team in Guangdong Modern Agricultural Research System (No. 2020KJ126), and Guangdong Province Rural Revitalization Strategy Special Project (No. 200-2018-XMZC-0001-107-0145).

## Conflict of Interest

The authors declare that the research was conducted in the absence of any commercial or financial relationships that could be construed as a potential conflict of interest.

## Publisher's Note

All claims expressed in this article are solely those of the authors and do not necessarily represent those of their affiliated organizations, or those of the publisher, the editors and the reviewers. Any product that may be evaluated in this article, or claim that may be made by its manufacturer, is not guaranteed or endorsed by the publisher.

## References

[1] Wang N Zhao D Wang J Zhang Y Wang M Gao Y. Architecture of African swine fever virus and implications for viral assembly. Science. (2019) 366:40–4. 10.1126/science.aaz1439 31624094

[2] Zhou X Li N Luo Y Liu Y Miao F Chen T. Emergence of African swine fever in China, 2018. Transbound Emerg Dis. (2018) 65:1482–4. 10.1111/tbed.12989 30102848

[3] Zhao D Liu R Zhang X Li F Wang J Zhang J. Replication and virulence in pigs of the first African swine fever virus isolated in China. Emerg Microbes Infect. (2019) 8:438–47. 10.1080/22221751.2019.1590128 30898043 PMC6455124

[4] Yoo DS Kim Y Lee ES Lim JS Hong SK Lee IS. Transmission dynamics of African swine fever virus, South Korea, 2019. Emerg Infect Dis. (2021) 27:1909–18. 10.3201/eid2707.204230 34152953 PMC8237864

[5] Dharmayanti NLPI Sendow I Ratnawati A Settypalli TBK Saepulloh M Dundon WG. African swine fever in North Sumatra and West Java provinces in 2019 and 2020, Indonesia. Transbound Emerg Dis. (2021) 68:2890–6. 10.1111/tbed.14070 33725423

[6] Truong QL Nguyen TL Nguyen TH Shi J Vu HLX Lai TLH. Genome sequence of a virulent African swine fever virus isolated in 2020 from a domestic pig in Northern Vietnam. Microbiol Resour Announc. (2021) 10:e00193–21. 10.1128/MRA.00193-21 33986078 PMC8142564

[7] Sun E Zhang Z Wang Z He X Zhang X Wang L. Emergence and prevalence of naturally occurring lower virulent African swine fever viruses in domestic pigs in China in 2020. Sci China Life Sci. (2021) 64:752–65. 10.1007/s11427-021-1904-4 33655434

[8] Chen W Zhao D He X Liu R Wang Z Zhang X. A seven-gene-deleted African swine fever virus is safe and effective as a live attenuated vaccine in pigs. Sci China Life Sci. (2020) 63:623–34. 10.1007/s11427-020-1657-9 32124180 PMC7223596

[9] Monteagudo PL Lacasta A Lopez E Bosch L Collado J Pina-Pedrero S. BA71 delta CD2: a new recombinant live attenuated African swine fever virus with cross-protective capabilities. J Virol. (2017) 91:e010558–17. 10.1128/JVI.01058-17 28814514 PMC5640839

[10] Gimenez-Lirola LG Mur L Rivera B Mogler M Sun Y Lizano S. Detection of African swine fever virus antibodies in serum and oral fluid specimens using a recombinant protein 30 (*p* 30) dual matrix indirect ELISA. PLoS ONE. (2016) 11:e0161230. 10.1371/journal.pone.0161230 27611939 PMC5017782

[11] Zhu Y Shao N Chen J Qi W Li Y Liu P. Multiplex and visual detection of African swine fever virus (ASFV) based on hive-chip and direct loop-mediated isothermal amplification. Anal Chim Acta. (2020) 1140:30–40. 10.1016/j.aca.2020.10.011 33218487 PMC7542229

[12] Chen D Wang D Wang C Wei F Zhao H Lin X. Application of an AlphaLISA method for rapid sensitive detection of African swine fever virus in porcine serum. Appl Microbiol Biot. (2021) 105:4751–9. 10.1007/s00253-021-11339-2 34050784

[13] Rai A Pruitt S Ramirez-Medina E Vuono EA Silva E Velazquez-Salinas L. Detection and quantification of African swine fever virus in MA-104 cells. Bio-Protocol. (2021) 11:e3955. 10.21769/BioProtoc.3955 33855107 PMC8032485

[14] Aira C Ruiz T Dixon L Blome S Rueda P Sastre P. Bead-based multiplex assay for the simultaneous detection of antibodies to African swine fever virus and classical swine fever virus. Front Vet Sci. (2019) 6:306. 10.3389/fvets.2019.00306 31572739 PMC6753221

[15] Kuypers J Jerome KR. Applications of digital PCR for clinical microbiology. J Clin Microbiol. (2017) 55:1621–8. 10.1128/JCM.00211-17 28298452 PMC5442518

[16] Wu X Xiao L Lin H Chen S Yang M An W. Development and application of a droplet digital polymerase chain reaction (ddPCR) for detection and investigation of African swine fever virus. Can J Vet Res. (2018) 82:70–4. 29382972 PMC5764039

[17] Jia R Zhang G Liu H Chen Y Zhou J Liu Y. Novel application of nanofluidic chip digital PCR for detection of African swine fever virus. Front Vet Sci. (2021) 7:621840. 10.3389/fvets.2020.621840 33614757 PMC7894257

[18] Gao Q Yang Y Quan W Zheng J Luo Y Wang H. The African swine fever virus with MGF360 and MGF505 deleted reduces the apoptosis of porcine alveolar macrophages by inhibiting the NF-κB signaling pathway and interleukin-1β. Vaccines. (2021) 9:1371. 10.3390/vaccines9111371 34835302 PMC8622997

[19] Wang X Ji P Fan H Dang L Wan W Liu S. CRISPR/Cas12a technology combined with immunochromatographic strips for portable detection of African swine fever virus. Commun Biol. (2020) 3:62. 10.1038/s42003-020-0796-5 32047240 PMC7012833

[20] Koltsova G Koltsov A Krutko S Kholod N Tulman ER Kolbasov D. Growth kinetics and protective efficacy of attenuated ASFV strain congo with deletion of the EP402 gene. Viruses. (2021) 13:1259. 10.3390/v13071259 34203302 PMC8309992

[21] Petrovan V Rathakrishnan A Islam M Goatley LC Moffat K Sanchez-Cordon PJ. Role of African swine fever virus (ASFV) proteins EP153R and EP402R in reducing viral persistence in blood and virulence in pigs infected with BeninDeltaDP148R. J Virol. (2021) 96:e0134021. 10.1128/JVI.01340-21 34643433 PMC8754224

[22] Ha TTT Anh KD Duc Viet L Hao TV Tuan VH Chinh TN. An improvement of real-time polymerase chain reaction system based on probe modification is required for accurate detection of African swine fever virus in clinical samples in Vietnam. Asian Austral J Anim. (2020) 33:1683–90. 10.5713/ajas.19.0525 32054190 PMC7463087

[23] Wang D Yu J Wang Y Zhang M Li P Liu M. Development of a real-time loop-mediated isothermal amplification (LAMP) assay and visual LAMP assay for detection of African swine fever virus (ASFV). J Virol Methods. (2020) 276:113775. 10.1016/j.jviromet.2019.113775 31726114

[24] Wu J Mukama O Wu W Li Z Habimana JDD Zhang Y. A CRISPR/Cas12a based universal lateral flow biosensor for the sensitive and specific detection of African swine-fever viruses in whole blood. Biosensors-Basel. (2020) 10:203. 10.3390/bios10120203 33321741 PMC7763806

[25] Fan X Li L Zhao Y Liu Y Liu C Wang Q. Clinical validation of two recombinase-based isothermal amplification assays (RPA/RAA) for the rapid detection of african swine fever virus. Front Microbiol. (2020) 11:1696. 10.3389/fmicb.2020.01696 32793160 PMC7385304

[26] Pinheiro LB Coleman VA Hindson CM Herrmann J Hindson BJ Bhat S. Evaluation of a droplet digital polymerase chain reaction format for DNA copy number quantification. Anal Chem. (2012) 84:1003–11. 10.1021/ac202578x 22122760 PMC3260738

[27] Lin Y Cao C Shi W Huang C Zeng S Sun J. Development of a triplex real-time PCR assay for detection and differentiation of gene-deleted and wild-type African swine fever virus. J Virol Methods. (2020) 280:113875. 10.1016/j.jviromet.2020.113875 32333943

[28] Guo Z Li K Qiao S Chen XX Deng R Zhang G. Development and evaluation of duplex TaqMan real-time PCR assay for detection and differentiation of wide-type and MGF505-2R gene-deleted African swine fever viruses. BMC Vet Res. (2020) 16:428. 10.1186/s12917-020-02639-2 33167979 PMC7654620

[29] Wang Z Li P Lin X Jia H Jiang Y Wang X. Application of portable real-time recombinase-aided amplification (rt-RAA) assay in the clinical diagnosis of ASFV and prospective DIVA diagnosis. Appl Microbiol Biot. (2021) 105:3249–64. 10.1007/s00253-021-11196-z 33835201

[30] Zhu X Su S Fu M Peng Z Wang D Rui X. A density-watershed algorithm (DWA) method for robust, accurate and automatic classification of dual-fluorescence and four-cluster droplet digital PCR data. Analyst. (2019) 144:4757–71. 10.1039/c9an00637k 31290860

