# Supplementary material for: Development and Application of a Duplex Droplet Digital Polymerase Chain Reaction Assay for Detection and Differentiation of EP402R-Deleted and Wild-Type African Swine Fever Virus
Source: Front Vet Sci. 2022 Jun 6;9:905706. doi: 10.3389/fvets.2022.905706 (PMC9207387; doi:10.3389/fvets.2022.905706)
Supplement: Supplementary file 1 [file Table_1.DOCX]

**TABLE S1** Detection of clinical samples by established duplex ddPCR assay

| Sample number | ddPCR assay (copies/μL) | | Real-time PCR assay  (Ct value) | |
| --- | --- | --- | --- | --- |
|  | EP402R | B646L | EP402R | B646L |
|  | 1708 | 1742 | 25.35 | 24.06 |
|  | 3240 | 3340 | 23.33 | 22.3 |
|  | 554 | 578 | 26.92 | 25.51 |
|  | 4300 | 4650 | 23.42 | 22.34 |
|  | 2700 | 2880 | 24.42 | 23.48 |
|  | 124 | 171 | 32.17 | 35.87 |
|  | 48 | 245 | 32.8 | 34.85 |
|  | 6510 | 7300 | 17.68 | 17.48 |
|  | 15.8 | 31.7 | NA | NA |
|  | 3520 | 3700 | 22.17 | 22.17 |
|  | 6970 | 5350 | 21.99 | 21.57 |
|  | 7280 | 5560 | 21.14 | 21.57 |
|  | 5680 | 5230 | 21.64 | 21.74 |
|  | 25.6 | 33.4 | 33.96 | 36.84 |
|  | 11.5 | 34.4 | NA | NA |
|  | 512 | 513 | 31.56 | 31.25 |
|  | 17.7 | 62.2 | NA | 33.37 |
|  | 650 | 667 | 26.72 | 25.46 |
|  | 11 | 10.3 | 27.09 | 32.01 |
|  | 60 | 39.3 | 25.03 | 30.49 |
|  | 23 | 23 | 26.32 | 31.44 |
|  | 5710 | 5770 | 17.88 | 20.85 |
|  | 2390 | 1967 | 19.54 | 24.22 |
|  | 78 | 25.1 | 27.69 | 34.70 |
|  | 174 | 143 | 24.24 | 28.53 |
|  | 2480 | 2361 | 19.30 | 24.05 |
|  | 7220 | 7370 | 17.27 | 21.55 |
|  | 8300 | 8800 | 17.50 | 21.11 |
|  | 8900 | 9700 | 16.96 | 20.84 |
|  | 447 | 494 | 24.15 | 27.02 |
|  | 4900 | 6100 | 17.18 | 21.62 |
|  | 78 | 82.3 | 27.30 | 30.55 |
|  | 13.9 | 7.9 | 27.46 | 32.89 |
|  | 3220 | 4440 | 24.61 | 23.20 |
|  | 7.9 | 9.1 | 28.16 | 33.32 |
|  | 26.1 | 12.5 | 28.08 | 33.03 |
|  | 358 | 353 | 25.29 | 28.03 |
|  | 5700 | 5980 | 17.79 | 22.17 |
|  | 455 | 329 | 22.98 | 27.61 |
|  | 486 | 435 | 21.87 | 26.23 |
|  | 13.1 | 11.5 | 30.43 | 35.72 |
|  | 67.8 | 95 | 28.50 | 29.42 |
|  | 5370 | 5180 | 22.43 | 23.12 |
|  | 4290 | 4240 | 21.58 | 23.16 |
| ∆EP402R-ASFV | 0 | 942 | NA | 25.41 |
| ∆EP402R/∆MGF360-505R-ASFV | 0 | 52.4 | NA | 30.22 |
| ∆MGF360-505R-ASFV | 549 | 627 | 23.86 | 26.15 |
| GZ201801-ASFV | 6380 | 5600 | 18.82 | 18.62 |
| water | 0 | 0.06 | NA | NA |

*Note.*“NA” means no Ct value was detected.
